# Laboratory challenges of Plasmodium species identification in Aceh Province, Indonesia, a malaria elimination setting with newly discovered *P*. *knowlesi*

**DOI:** 10.1371/journal.pntd.0006924

**Published:** 2018-11-30

**Authors:** Farah N. Coutrier, Yusrifar K. Tirta, Chris Cotter, Iska Zarlinda, Iveth J. González, Alanna Schwartz, Cut Maneh, Jutta Marfurt, Maxwell Murphy, Herdiana Herdiana, Nicholas M. Anstey, Bryan Greenhouse, Michelle S. Hsiang, Rintis Noviyanti

**Affiliations:** 1 Malaria Pathogenesis Unit, Eijkman Institute for Molecular Biology, Jakarta, Indonesia; 2 Malaria Elimination Initiative, Global Health Group, University of California, San Francisco, San Francisco, California, United States of America; 3 Foundation for Innovative New Diagnostics, Geneva, Switzerland; 4 Department of Medicine, University of California, San Francisco, San Francisco, California, United States of America; 5 Unit Pelaksana Teknis Dinas Laboratorium Kesehatan Daerah, Banda Aceh, Indonesia; 6 Menzies School of Health Research, Darwin, Australia; 7 Paritrana Asia Foundation, Jakarta, Indonesia; 8 United Nations Children’s Fund, Aceh Field Office, Banda Aceh, Indonesia; 9 Department of Pediatrics, University of Texas Southwestern Medical Center, Dallas, Texas, United States of America; 10 Department of Pediatrics, University of California, San Francisco, Benioff Children’s Hospital, San Francisco, California, United States of America; Walter and Eliza Hall Institute, AUSTRALIA

## Abstract

The discovery of the life-threatening zoonotic infection *Plasmodium knowlesi* has added to the challenges of prompt and accurate malaria diagnosis and surveillance. In this study from Aceh Province, Indonesia, a malaria elimination setting where *P*. *knowlesi* endemicity was not previously known, we report the laboratory investigation and difficulties encountered when using molecular detection methods for quality assurance of microscopically identified clinical cases. From 2014 to 2015, 20 (49%) *P*. *falciparum*, 16 (39%) *P*. *vivax*, 3 (7%) *P*. *malariae*, and 2 (5%) indeterminate species were identified by microscopy from four sentinel health facilities. At a provincial-level reference laboratory, loop-mediated isothermal amplification (LAMP), a field-friendly molecular method, was performed and confirmed Plasmodium in all samples though further species-identification was limited by the unavailability of non-falciparum species-specific testing with the platform used. At a national reference laboratory, several molecular methods including nested PCR (nPCR) targeting the 18 small sub-unit (18S) ribosomal RNA, nPCR targeting the cytochrome*-b (cytb)* gene, a *P*. *knowlesi*-specific nPCR, and finally sequencing, were necessary to ultimately classify the samples as: 19 (46%) *P*. *knowlesi*, 8 (20%) *P*. *falciparum*, 14 (34%) *P*. *vivax*. Microscopy was unable to identify or mis-classified up to 56% of confirmed cases, including all cases of *P*. *knowlesi*. With the nPCR methods targeting the four human-only species, *P*. *knowlesi* was missed (18S rRNA method) or showed cross-reactivity for *P*. *vivax* (*cytb* method). To facilitate diagnosis and management of potentially fatal *P*. *knowlesi* infection and surveillance for elimination of human-only malaria in Indonesia and other affected settings, new detection methods are needed for testing at the point-of-care and in local reference laboratories.

## Author summary

In Southeast Asia, *Plasmodium knowlesi*, a malaria parasite of macaques, was recently discovered to infect humans. This emerging disease is important because it has potential for causing severe disease and death, and it is a threat to malaria elimination efforts in the region. In this report from Aceh Province, Indonesia, where *P*. *knowlesi* was only recently discovered, the authors report on the laboratory challenges of distinguishing this species from other human species. Using several different molecular methods, they investigated 41 malaria cases which by microscopy, were initially reported as: *P*. *falciparum* (49%), *P*. *vivax* (39%), *P*. *malariae* (7%), and indeterminate (5%). Only after using a *P*. *knowlesi*-specific nPCR method and sequencing, did they find that nearly half were *P*. *knowlesi*. Consistent with a sparse literature, a field-friendly molecular method (genus-specific LAMP) reliably detected *P*. *knowlesi*, while use of a more standard reference laboratory molecular method (18S rRNA nPCR targeting the four human-only species) missed the infections. Also another reference laboratory molecular method (*cytb* nPCR) mis-classified *P*. *knowlesi* infections as *P*. *vivax* due to cross-reactivity. To address the emerging threat of *P*. *knowlesi*, new detection methods are needed for point-of-care and reference testing.

## Introduction

*Plasmodium knowlesi* is a newly emergent zoonotic human malaria species previously thought to only infect macaques. Since the first report of a human case from Peninsular Malaysia in 1965 [1] and the large cluster of human knowlesi malaria in Sarawak in 2004 [2], endemic cases have been reported from other Asian countries including Brunei, Cambodia, India, Malaysia, Myanmar, Philippines, Singapore, Thailand, Vietnam, Indonesian Borneo [3–5], and more recently Sumatra Island [6, 7].

The identification of *P*. *knowlesi* infection is important for clinical and public health reasons. Infection in humans is most often uncomplicated, but 6–9% of symptomatic patients develop severe malaria and 0.3–1.8% of cases die [8–10]. Fatal outcomes have been associated with misdiagnosis of parasite species by microscopy, resulting in delays in appropriate management [11, 12]. From a public health perspective, malaria control programs aim to decrease morbidity and mortality from all Plasmodium species affecting humans. As *P*. *knowlesi* infection is associated with a number of different risk factors than infections caused by other Plasmodium species [6, 13] (e.g. forest-related exposures), it may require different interventions. For subnational and national areas aiming to achieve and maintain malaria elimination, or the interruption of local transmission of human-only species, as is the goal in Indonesia, accurate species identification is critical.

In most of Asia, microscopy is the standard for malaria diagnosis and surveillance. However microscopy has recognized limitations in diagnostic accuracy and species identification [14]. For *P*. *knowlesi* specifically, different asexual blood stages can resemble *P*. *falciparum* and *P*. *malariae*, and in routine practice it is misidentified as all human-only species [15]. Therefore, a variety of PCR methods have been utilized to distinguish *P*. *knowlesi* from other Plasmodium species [16, 17]. With its simpler requirements and faster turnaround time, loop mediated isothermal amplification (LAMP), another nucleic acid-based detection method, may be a more practical alternative in resource-limited field settings [18–20]. However, the relative benefits and limitations of LAMP and the various other PCR methods are not clear, particularly for field settings.

To support malaria elimination efforts in Aceh Province, Indonesia, a pre-elimination area with known endemicity of *P*. *vivax* and *P*. *falciparum*, we introduced the use of molecular detection for quality assurance of microscopy-identified cases from health facilities by establishing LAMP testing at the provincial level reference laboratory. As previously reported, the finding of indeterminate species triggered further molecular testing that led to the first reported finding of *P*. *knowlesi* in Indonesia outside of Borneo [6]. Epidemiological investigation revealed that *P*. *knowlesi* infection was associated with forest exposures, particularly overnight stays due to work [6].

In this study, we present the laboratory details of this real-world investigation whereby the use of serial molecular detection methods including LAMP, two nPCR methods, *P*. *knowlesi*-specific nPCR, and sequencing led to the identification and confirmation of *P*. *knowlesi* infection. Challenges encountered in this experience have relevance to malaria diagnosis and surveillance in other settings where *P*. *knowlesi* may be present and can inform research and development of improved *P*. *knowlesi* detection methods.

## Materials and methods

### Study site and patient enrollment

The study was conducted in Aceh Besar District, Aceh Province, Sumatra island, Indonesia, a low-transmission setting that aims to eliminate malaria by 2020. The 2013 incidence of malaria was 0.4/1000, and 68 (39%) of cases were reported as *P*. *vivax*, 71 (41%) as *P*. *falciparum*, and the remaining 34 unspecified or mixed *P*. *falciparum*/*P*. *vivax* [6]. The sentinel sites included five primary health centers that reported 78% of all cases reported in Aceh Besar in 2013. During the study period June 2014 to December 2015, 41 patients were diagnosed with microscopy-confirmed malaria and recruited for enrolment. This number of cases was a convenience sample from an umbrella study where health facility-identified cases triggered active case finding in villages [6].

After written consent was obtained and prior to treatment, venous blood was collected and partly used to prepare dried blood spots (DBS) using Whatman 3MM paper. DBS along with remaining whole blood were initially stored at 4°C, transferred to -20°C within a week of collection, and then stored at -80°C. Antimalarial treatment was based on microscopy results and according to Indonesian government’s national policy.

### Ethical approval

Ethical approval for the study was obtained from the National Institute of Health, Research and Development of the Indonesian Ministry of Health (number LB.02.01/5.2/KE.111/2014 and LB.02.01/5.2/KE.211/2015) and IRB Committee of the University of California, San Francisco. Written informed consent was obtained from all adults or a parent or guardian for participants less than 18 years of age.

### Laboratory methods

For quality assurance of microscopy performed at health centers, blood smears were re-read by certified microscopists at the provincial laboratory according to national guidelines. For further quality assurance at the provincial-level, LAMP was selected due to its field-friendly platform. Initial extraction of DNA and LAMP testing were performed at the provincial laboratory. DNA was extracted from DBS using the Saponin/Chelex method [21]. Pan-LAMP testing followed by Pf-LAMP specific testing for Pan-LAMP positive samples was also performed using the commercially available Loopamp MALARIA Pan/Pf detection kit in accordance to manufacturer’s instructions (EIKEN Chemical, Co., Ltd., Japan). Species identification for non-falciparum species was not available with this LAMP platform, but this was not anticipated to be a problem because Aceh was considered to be endemic for only *P*. *falciparum* and *P*. *vivax* malaria before the study was launched [22]. As such, Pan-LAMP positive, Pf-LAMP negative samples were expected to be *P*. *vivax*.

Further molecular testing was performed at the Malaria Pathogenesis laboratory at the Eijkman Institute in Jakarta, using chelex-extracted DNA from a second DBS. Genus-specific PCR targeting the mitochondrial *cytb* gene followed by *Alu*I enzyme digestion for species identification of the four main human species was used initially, as previously described [23]. After a report of indeterminate species and suspicion of *P*. *knowlesi* by a field microscopist, as well as limited data on the performance of the *cytb* nPCR method for detection of *P*. *knowlesi*, additional methods were employed including nPCR testing targeting the 18S rRNA gene for the four human-only species [24], and *P*. *knowlesi*-specific nPCR [16] for all samples. For a proportion of samples testing positive by *P*. *knowlesi* specific nPCR, DNA was extracted from whole blood using the QIAamp DNA Mini kit (Qiagen, CA) and Sanger targeted genome sequencing [25] was performed (Eijkman Institute Sequencing Facility). To prevent DNA contamination, all extractions were performed in rooms separate from where amplification was conducted. Extracted DNA was stored at -20°C.

### Data analysis

Results from microscopy and each molecular method were compared to a gold standard established through serial molecular testing: *P*. *falciparum* and *P*. *vivax* classification were based on species-specific positivity by both *cytb* and 18S rRNA nPCR, and *P*. *knowlesi* classification was based on genus-specific PCR positivity by both *cytb* and 18S rRNA nPCR and *P*. *knowlesi*-specific nPCR positivity. With regards to diagnostic performance for species identification, we were not able to calculate sensitivity, specificity, or negative predictive value (NPV) due to having not included a representative sample of microscopy-negative infections. However, positive predictive values (PPV) were calculated.

## Results

### Enrollment and microscopy results

**Fig 1:**
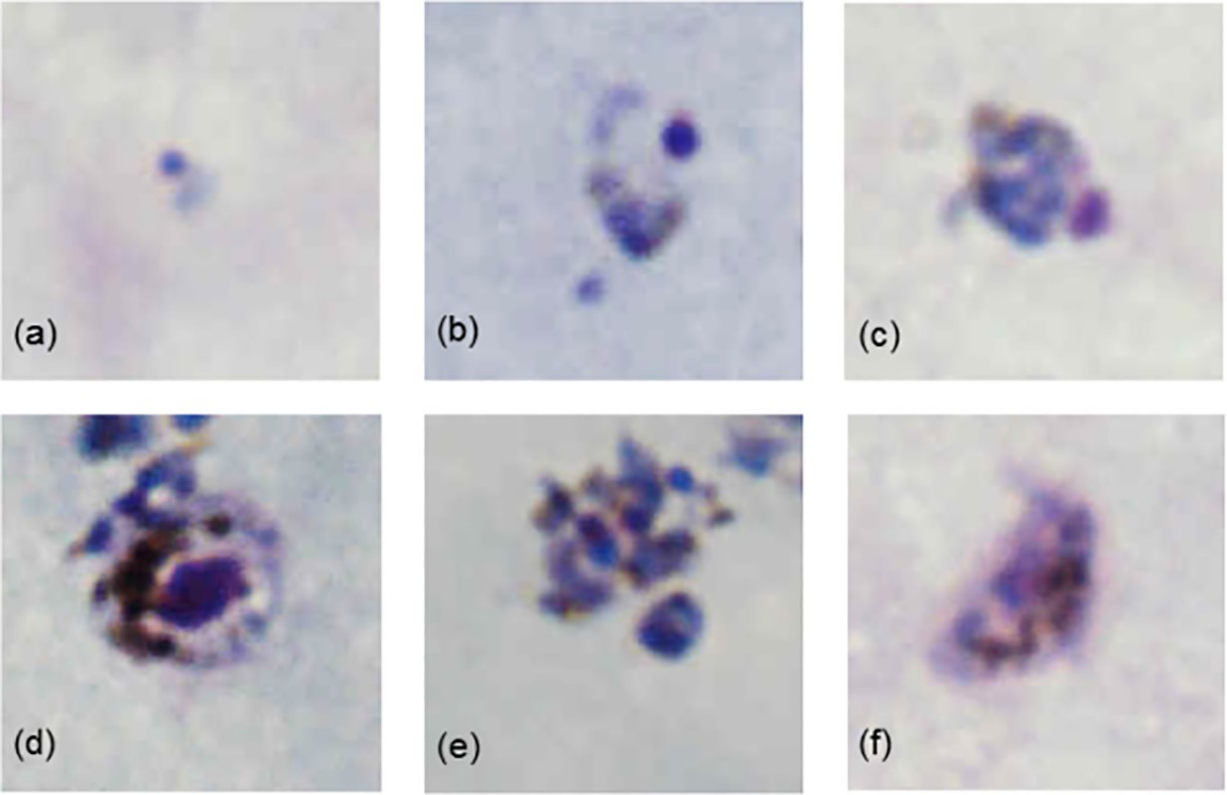
Microscopy images from samples initially classified as indeterminate but later confirmed to be *P*. *knowlesi*. (a) early trophozoite resembling *P*. *falciparum*; (b) trophozoite resembling *P*. *vivax*; (c and d) late trophozoite resembling *P*. *malariae*; (e) multi-nucleated schizont and (f) gametocyte resembling *P*. *falciparum*.

From June 2014 to December 2015, 41 malaria cases were included in the study analysis. Forty-two were initially identified from the sentinel health facilities by microscopy and confirmed by cross-checking at the provincial laboratory, but one case (*P*. *vivax* by microscopy) was excluded as the DBS had insufficient blood for subsequent molecular testing. The 41 cases included: 20 *P*. *falciparum* (49%), 16 *P*. *vivax* (39%), 3 *P*. *malariae* (7%), and 2 with indeterminate morphology (5%) (Table 1). Parasite density ranged from 66 to 355,400 parasite/μL blood. The median and range of parasite density (in brackets) for microscopy-diagnosed *P*. *falciparum*, *P*. *vivax* and *P*. *malariae* were 5,447 (66 to 54,970), 32,157 (703 to 355,400) and 3,842 (1,760 to 7,133). The parasite densities of the indeterminate samples were 803 and 1,473, respectively. Microphotography of the indeterminate samples showed resemblance to other species (Fig 1).

**Table 1:** Species classification of microscopy-positive samples by loop mediated isothermal amplification (LAMP), *cytb* nPCR, 18S rRNA *n*PCR, *Plasmodium knowlesi*-specific nPCR, and the serial molecular testing as gold standard.

Gold standard		Microscopy				LAMP		*cytb* nPCR		18S rRNA nPCR			Pk nPCR
		Pf	Pv	Pm	IND	Pan	Pf	Pf	Pv	Pf	Pv	Neg*	Pk
Pf	8 (19%)	8	-	-	-	8	8	8	-	8	-	-	-
Pv	14 (33%)	4	10	-	-	14	-	-	14	-	14	-	-
Pm	0 (0%)	-	-	-	-	-	-	-	-	-	-	-	-
Pk	19 (45%)	8	6	3	2	19	1	-	19	-	-	19	19
Total	41	20	16	3	2	41	9	8	33	8	14	19	19

LAMP: loop mediated isothermal amplification; Pf: *Plasmodium falciparum*; Pv: *P*. *vivax*

Pm: *P*. *malariae*; Pk: *P*. *knowlesi*; IND: indeterminate; Neg: negative; Pan: Pan-species.

*No amplification with primers targeting the four human-only species Pf, Pv, Pm, and Po

### Molecular testing results

**Fig 2:**
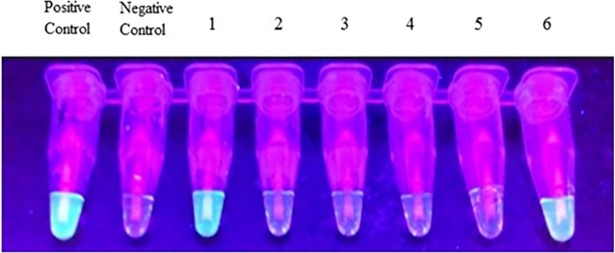
Loop mediated isothermal amplification (LAMP) detection of malaria. Pan-LAMP accurately identified malaria positive samples, later confirmed as *P*. *vivax* (tube 1) and *P*. *knowlesi* (tube 6).

Genus-specific Pan-LAMP testing at the provincial laboratory was positive in all 41 isolates (examples in Fig 2), and 8 tested positive by Pf-LAMP testing (Table 1). By *cytb* PCR genus-specific testing and using the *Alu*I restriction digest reaction for species identification, 8 (19.5%) were classified as *P*. *falciparum*, 33 (80.5%) as *P*. *vivax*. By 18S rRNA nPCR, there were 8 *P*. *falciparum* (19.5%), 14 *P*. *vivax* (34.1%), and 19 (46.3%) did not amplify. *P*. *knowlesi*-specific nPCR was positive in 19/41 (46.3%), of which 11 underwent sequencing and showed 100% identity to a published *P*. *knowlesi* 18S rRNA gene sequence (*P*. *knowlesi* strain H1 chromosome 3, GenBank accession number AM910985).

### Mis-classification or missing species identification

Microscopy was unable to classify or mis-classified 23 of 41 (56%) malaria cases confirmed by the gold standard of serial molecular testing (Table 1). These included all 19 *P*. *knowlesi* cases, of which 17 were mis-classified as *P*. *falciparum* (n = 8), *P*. vivax (n = 6), or *P*. *malariae* (n = 3), and 2 were unable to be classified. There were also 4 *P*. *vivax* cases that were mis-classified as *P*. *falciparum* by microscopy. Sixty percent (12/20) of cases identified by microscopy as *P*. *falciparum* were either *P*. *vivax* or *P*. *knowlesi*; 37.5% (6/16) of cases identified by microscopy as *P*. *vivax* were *P*. *knowlesi*. All *P*. *malariae* and indeterminate species by microscopy were *P*. *knowlesi*.

Genus-specific testing by LAMP identified all infections, though species identification was limited by the unavailability of non-falciparum species-specific testing with the platform used. Pf-LAMP testing mis-classified one *P*. *knowlesi* mono-infection as *P*. *falciparum* but otherwise correctly identified all the *P*. *falciparum* cases.

**Fig 3:**
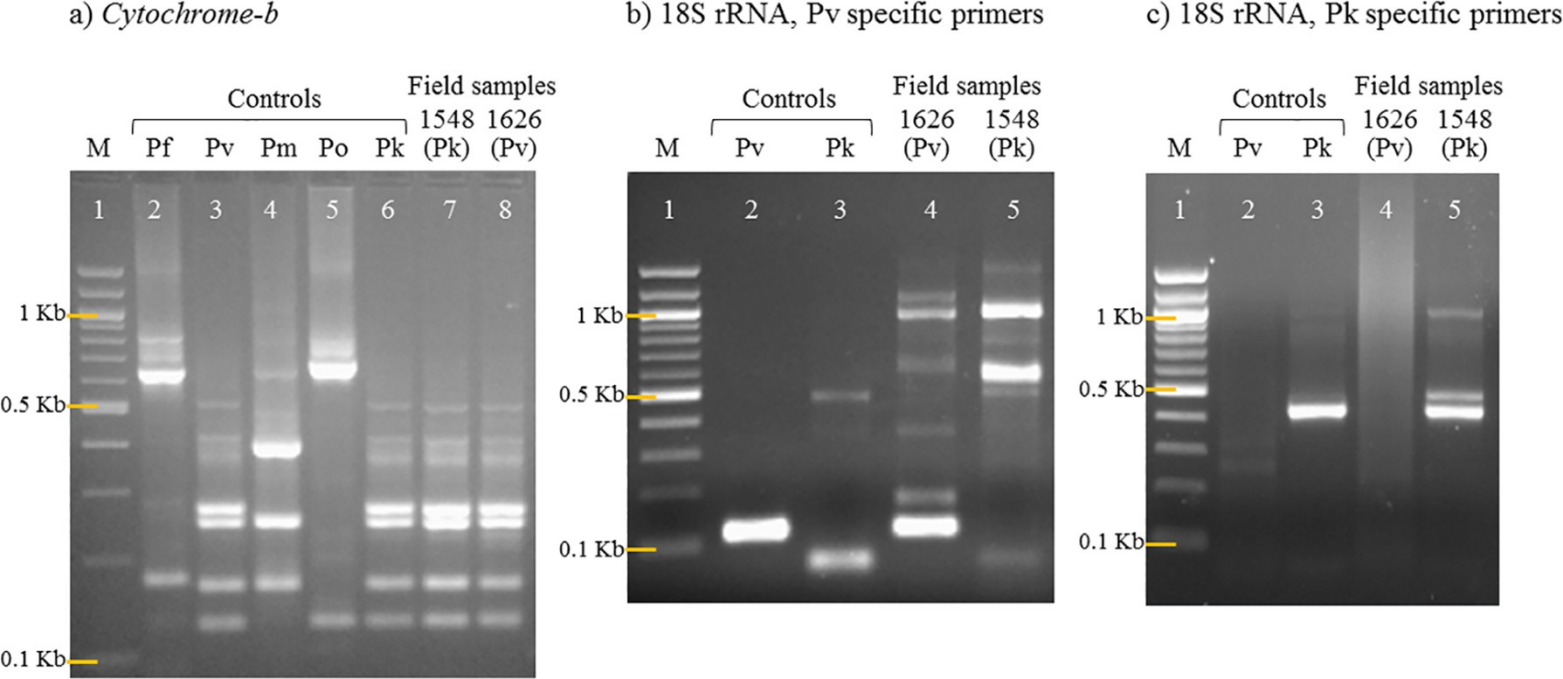
Mis-classification or missed malaria species identification using standard PCR. a) *Alu*I digestion of *cytochrome-b* nPCR product for species determination [23]. Pk control (lane 6) with similar banding pattern as Pv control (lane 3). Pk field sample 1548 (lane 7) incorrectly classified as Pv. Pv field sample 1626 (lane 8) accurately identified as Pv; b) 18S rRNA nPCR results utilizing Pv specific primers [24]. Pv field sample 1626 (lane 4) accurately identified as Pv and Pk field sample 1548 (lane 5) not successfully amplified; c) Pk-specific nPCR [16]. Successful amplification of Pk field sample 1548 but not Pv field sample 1626. M: Molecular weight marker; Pf: *Plasmodium falciparum*; Pv: *P*. *vivax*; Pm: *P*. *malariae*; Po: *P*. *ovale;* Pk: *P*. *knowlesi*.

Of cases classified as *P*. *vivax* by *cytb* PCR, 58% (19/33) were later confirmed as *P*. *knowlesi* and showed a similar banding pattern to *P*. *vivax* (Table 1 and Fig 3A). Using 18S rRNA species-specific nPCR for the four main human species, *P*. *falciparum* and *P*. *vivax* were correctly identified but all *P*. *knowlesi* infections were missed (Fig 3B). There was no cross-reactivity with *P*. *vivax* using *P*. *knowlesi*-specific nPCR (Fig 3C).

The positive predictive values (PPV) for species identification by different diagnostic methods using the gold standard of serial molecular testing are shown in Table 2. PPV was low for *P*. *falciparum*, *P*. *vivax*, and *P*. *malariae* identification by microscopy and for *P*. *vivax* identification by *cytb* nPCR. Where samples were available, PPV was high for all other methods.

**Table 2:** Positive predictive values for species identification by different diagnostic methods using the gold standard of serial molecular testing.

	Microscopy N, % (95%CI)		LAMP N, % (95%CI)		*cytb* nPCR N, % (95%CI)		18S rRNA nPCR N, % (95%CI)		Pk nPCR N, % (95%CI)	
*P*. *falciparum*	20	40.0 (18.5–61.5)	9	88.9 (68.4–100)	8	100	8	100	-	-
*P*. *vivax*	16	62.5 (38.8–86.2)	-	-	33	42.4 (25.6–59.3)	14	100	-	-
*P*. *malariae*	3	0	-	-	-	-	-	-	-	-
*P*. *knowlesi*	-	-	-	-	-	-	-	-	19	100

## Discussion

To support malaria diagnosis and surveillance in Aceh Province, a low transmission setting in Indonesia that is aiming for malaria elimination, we utilized molecular testing for quality assurance of microscopy-confirmed cases from health facilities. As previously published, this work resulted in the first report of *P*. *knowlesi* in Indonesia outside Borneo, and an epidemiological investigation revealed that forest exposures are a key risk factor for this zoonotic infection [6]. In this study, we report the details and difficulties of species identification using microscopy at the point of care and a variety of molecular methods at reference laboratories. Microscopy mis-classified *P*. *knowlesi* cases as *P*. *malariae* or *P*. *falciparum*, as commonly reported elsewhere, but also as *P*. *vivax*, which has been less commonly reported [15]. The PPVs for the identification of other species (Pf, Pv, and Pm) were also poor. At the provincial reference laboratory, LAMP, a field-friendly molecular method, was useful in confirming all Plasmodium infections, though further species identification was limited by the unavailability of non-falciparum species-specific testing with the platform used. Use of less field-friendly nPCR methods at a national reference laboratory to identify *P*. *knowlesi* infection was not straightforward. All *P*. *knowlesi* cases did not amplify with a standard nPCR method (18S rRNA) targeting the four human-only species. With the *cytb* method, there was cross-reactivity with *P*. *vivax* for all *P*. *knowlesi* cases. We highlight the difficulties of *P*. *knowlesi* diagnosis at the point-of-care and reference laboratory levels in a setting where endemicity was not previously known and bring attention to an emerging challenge for malaria elimination.

The recent discovery and emergence of *P*. *knowlesi*, a fifth human species previously thought to only infect macaques, has created an additional challenge for species identification. Microscopy is difficult because the morphology at different stages resembles other malaria species [26]. The diagnostic sensitivity and specificity of available immunochromatographic rapid diagnostic tests (RDTs) for *P*. *knowlesi* detection is poor, leaving no other useful point-of-care diagnostic test [27–29]. Despite some global knowledge on the potential geographical distribution and extent of transmission of *P*. *knowlesi* [4], this information may lack resolution at local levels, and health workers and microscopists on the front-lines may have limited knowledge and/or a low index of suspicion for *P*. *knowlesi*. In our study, the investigation into *P*. *knowlesi* was prompted by the observation by an astute microscopist of unusual morphology in two malaria cases, as well as the known local presence of pig-tailed and long-tailed macaques and *Anopheles leucosphyrus*, a known vector on Sumatra island [30].

For quality control in reference laboratories, none of the nucleic acid-based methods for both genus and species-specific identification were found to be suitable. With LAMP, a molecular detection method that has been promoted for use in resource-limited settings due to the rapid turnaround time and simple methods, genus-specific testing was reliable, as has been reported from Malaysia [20]. However a *P*. *knowlesi-*specific commercial kit was not available for use in our study, and evaluations of other *P*. *knowlesi*-specific LAMP assays have reported cross-reactivity with *P*. *vivax* [18]. The *P*. *knowlesi*-specific PCR method utilized in this study did not cross-react with *P*. *vivax* infections, with excellent specificity as observed previously [16]. The nPCR methods used have problems with missed infections and/or species mis-classification. With commonly used 18S rRNA nPCR targeting the four human-only species, a commonly used reference standard, *P*. *knowlesi* either does not amplify (as occurred in this study) or is mis-classified as *P*. vivax due to high sequence homology at the target sequences [31, 32]. With the *cytb* nPCR method that we used, our finding of cross-reactivity between *P*. *knowlesi* and *P*. *vivax* has not been previously reported, but can also be explained by high sequence homology at the target mitochondrial sequences. Others have reported *P*. *knowlesi* amplification using *P*. *vivax*-specific PCR targeting the mitochondrial gene *cox1* [33]. Other more sensitive and specific molecular methods for *P*. *knowlesi* detection in mixed species settings have recently been developed [7, 34, 35] and could be considered for future surveillance in our study setting.

The challenge of accurate *P*. *knowlesi* detection is of both clinical and public health significance. In Malaysia, where the clinical disease has been well studied, *P*. *knowlesi* is associated with at least as high a risk of severe disease compared with *P*. *falciparum* [36] and in early series, a high proportion had fatal outcomes [8, 37]. Following a number of interventions in Sabah state, case-fatality rates have fallen 6-fold [9]. These have included improved and now routine statewide molecular surveillance, more recent laboratory microscopy reporting of “*P*. *malariae*” as “*P*. *knowlesi*”, and enhanced implementation of standardized referral and clinical protocols, including first-line use of artemisinin-based combination therapy and early intravenous artesunate [9, 36]. Progression to severe disease is due not only to missed diagnoses, but also its ability to cause severe malaria at relatively low parasite densities [36]. Mis-classification of *P*. *knowlesi* as *P*. *vivax*, as occurred at the point of care in our study, also results in unnecessary treatment with primaquine, an antimalarial not indicated for *P*. *knowlesi*, but necessary for radical cure of the latent liver stages with *P*. *vivax*. In our study, we did not experience any severe adverse events from the unnecessary use of primaquine, but use in subjects with underlying severe glucose-6-phosphate dehydrogenase deficiency is known to be associated with life-threatening hemolysis.

While only recently recognized in areas of Aceh and North Sumatra, there has been little molecular surveillance of *P*. *knowlesi* distribution and incidence elsewhere in Indonesia, particularly across Kalimantan, Sulawesi and other regions of Sumatra, where modelling predicts a high risk of human infection [38]. From a public health perspective, accurate identification of *P*. *knowlesi* is critical to the design and implementation of effective malaria interventions. In a related study in Aceh Province and also in Malaysia, adult males with forest-related and agricultural occupational exposure are at significantly higher risk of being infected with *P*. *knowlesi* [6, 13]. Interventions would therefore need to be targeted to this population. As well as continued promotion of conventional malaria prevention activities to reduce peridomestic transmission [13], other interventions would need to be targeted to *P*. *knowlesi*-transmitting mosquitos, the interface between humans and macaques, and to individual risk factors for infection identified in different settings. Further investigation into the epidemiology and transmission of *P*. *knowlesi* in Aceh Besar is needed.

## Conclusions

Limitations of microscopy to identify *P*. *knowlesi* are well established. Our challenges using LAMP and PCR for species identification in a setting with previously unknown *P*. *knowlesi* endemicity add to a growing literature on the limitations of molecular methods as well. For settings approaching malaria elimination and/or where epidemiological conditions are predicted to support *P*. *knowlesi* transmission to humans, quality assurance of malaria diagnosis and species identification is essential, but at present, practical and accurate methods are not available for local and peripheral reference laboratories. Development, evaluation and implementation of improved *P*. *knowlesi* detection methods for use at both the point-of-care and in local reference laboratories are needed.
